# The effects of sleep and wakefulness on human fear conditioning

**DOI:** 10.1192/j.eurpsy.2021.489

**Published:** 2021-08-13

**Authors:** Y. Pavlov, B. Kotchoubey

**Affiliations:** 1 Department Of Psychology, Ural Federal University, Ekaterinburg, Russian Federation; 2 Institute Of Medical Psychology, University of Tuebingen, Tuebingen, Germany

**Keywords:** Fear conditioning, EEG, ECG, sleep

## Abstract

**Introduction:**

Studies on fear conditioning have made important contributions to the understanding of affective learning mechanisms as well as its applications (e.g., anxiety disorders, post-traumatic stress disorder). However, central mechanisms of sleep related consolidation of fear memory in humans have been almost neglected by previous studies.

**Objectives:**

In the current study we aimed to test effects of sleep and a period wakefulness on fear conditioned responses.

**Methods:**

In our experiment in a group 18 healthy volunteers event-related brain potentials (ERP), heart rate variability (HRV) and behavioral responses were recorded during a fear conditioning procedure presented twice, before daytime sleep (2h) or control intervention (a period of wakefulness) and after. The conditioning procedure involved pairing of a neutral tone (CS+) with a highly unpleasant sound (UCS+).

**Results:**

Differential conditioning manifested itself in the contingent negative variance (CNV)-like slow ERP component. Both period of sleep and wakefulness resulted in an increased amplitude of the CNV to CS+. But we did not find an interaction effect of Time (Pre-Post) by Intervention (Sleep-Wake), suggesting that sleep did not affect the conditioned response differently as compared to a period of wakefulness. An apparent increase in HRV after a period of wakefulness did not affect fear conditioned responses (CNV and valence ratings).

**Conclusions:**

To summarize, the data indicate that fear memories are consolidated with the course of time with no beneficial effect of sleep; relearning of fear causes stronger differential responses as measured by slow wave amplitude but not behavior; increase of HRV does not affect fear learning.

